# Towards personalized mapping through lumbosacral spinal cord task fMRI

**DOI:** 10.1162/imag_a_00455

**Published:** 2025-01-23

**Authors:** Sergio Daniel Hernandez-Charpak, Nawal Kinany, Ilaria Ricchi, Raphaëlle Schlienger, Loan Mattera, Roberto Martuzzi, Bruno Nazarian, Robin Demesmaeker, Andreas Rowald, Anne Kavounoudias, Jocelyne Bloch, Grégoire Courtine, Dimitri Van De Ville

**Affiliations:** Defitech Center for Interventional Neurotherapies (.NeuroRestore), CHUV/UNIL/EPFL, Lausanne, Switzerland; Neuro-X Institute, Ecole Polytechnique Fédérale de Lausanne (EPFL), Geneva, Switzerland; Department of Clinical Neuroscience, Lausanne University Hospital (CHUV) and University of Lausanne (UNIL), Lausanne, Switzerland; Department of Radiology and Medical Informatics, University of Geneva, Geneva, Switzerland; Aix-Marseille Université, CNRS, Centre de Recherche en Psychologie et Neurosciences, CRPN UMR 7077, Marseille, France; Fondation Campus Biotech Genève, Geneva, Switzerland; Aix-Marseille Université, CNRS, Centre IRM-INT@CERIMED (Institut des Neurosciences de la Timone – UMR 7289), Marseille, France; Professorship of Digital Health, Department of Medical Informatics, Biometry and Epidemiology & Department Artificial Intelligence in Biomedical Engineering, Friedrich-Alexander-Universität Erlangen-Nürnberg, Erlangen, Germany; Faculty of Biology and Medicine, University of Lausanne, Lausanne, Switzerland; Department of Neurosurgery, Lausanne University Hospital (CHUV) and University of Lausanne (UNIL), Lausanne, Switzerland

**Keywords:** fMRI, task, spinal cord, lumbosacral, muscle

## Abstract

The lumbosacral spinal cord contains neural circuits crucial for locomotion, organized into rostrocaudal levels with distinct somatosensory and motor neuron pools that project to and from the muscles of the lower limbs. However, the specific spinal levels that innervate each muscle and the locations of neuron pools vary significantly between individuals, presenting challenges for targeted therapies and neurosurgical interventions aimed at restoring locomotion. Non-invasive approaches to functionally map the segmental distribution of muscle innervation*—*or*projectome—*are therefore essential. Here, we developed a pipeline dedicated to record blood oxygenation level dependent (BOLD) signals in the lumbosacral spinal cord using functional magnetic resonance imaging (fMRI). We assessed spinal activity across different conditions targeting the extensor/flexor muscles of the right leg (ankle, knee, and hip) in 12 healthy participants. To enhance clinical relevance, we included not only active movements but also two conditions that did not rely on participants’ performance: passive stretches and muscle-specific tendon vibration, which activates proprioceptive afferents of the vibrated muscle. BOLD activity patterns were primarily located on the side ipsilateral to the movement, stretch, or vibration, both at the group and participant levels, indicating the BOLD activity being associated with the projectome. The fMRI-derived rostrocaudal BOLD activity patterns exhibited mixed alignment with expected innervation maps from invasive studies, varying by muscle and condition. While some muscles and conditions matched well across studies, others did not. Significant variability among individual participants underscores the need for personalized mapping of projections for targeted therapies and neurosurgical interventions. To support the interpretation of BOLD activity patterns, we developed a decision tree-based framework that combines reconstruction of neural structures from high-resolution anatomical MRI datasets and muscle-specific fMRI activity to produce personalized projectomes. Our findings provide a valuable proof-of-concept for the potential of fMRI to map the lumbosacral spinal cord’s functional organization, while shedding light on challenges associated with this endeavor.

## 1 Introduction

The spinal cord hosts ascending and descending white matter tracts that convey executive commands from the brain (*i.e.*, for motor control) and sensory information from peripheral organs (*i.e.*, for tactile and proprioceptive feedback). In addition, its gray matter contains distributed networks of neurons that are essential for the regulation of sensory, motor, and autonomic functions. Neurological conditions that alter the integrity of the spinal cord lead to broad impairments, with dramatic consequences for the lives of affected people and their family. Various treatments have been developed or are under development to improve the recovery of impaired neurological functions. Many of these treatments involve neuromodulation therapies, employing non-invasive methodologies or surgically implanted neurostimulation systems that aim to target precise neural structures and regions of the spinal cord (Kathe et al., 2022;Lorach et al., 2023;Milekovic et al., 2023;Rowald et al., 2022;Wagner et al., 2018). However, therapy design and clinical decision making of those treatments are currently limited by the absence of diagnostic tools that inform the functional organization of the spinal cord, in addition to the structural information.

The spinal cord follows a distinct somatotopic arrangement. Skeletal muscles are innervated by motor neuron pools that are organized segmentally along the rostrocaudal extent of the spinal cord. Sensory neurons are located in the dorsal root ganglions, from where they extend afferent projections through the dorsal roots to innervate the gray matter of the spinal cord. These projections not only carry sensory information to the brain via white matter tracts, they also establish transsynaptic connections to motor neurons (Bican et al., 2013).

Although anatomical studies have aimed at delineating the structural organization of the human spinal cord, these assessments are not sufficient to expose the functional organization of neuron pools innervating different muscles. At present, motor neuron pool distributions in humans have been primarily inferred from indirect clinical observations (Sharrard, 1964) or intraoperative experiments that linked the electrical stimulation of specific dorsal roots to responses in muscles in small cohorts of human participants (London et al., 2022;McIntosh et al., 2023;Phillips & Park, 1991;Schirmer et al., 2011). These intraoperative mappings have contributed to the construction of maps that describe the average distribution of somatosensory projections to motor neurons in the cervical (McIntosh et al., 2023;Schirmer et al., 2011) and lumbosacral (London et al., 2022;Phillips & Park, 1991;Schirmer et al., 2011) spinal cord. Although indirect clinical studies (Sharrard, 1964) have reported that somatosensory neuron projections associated with one muscle generally project through only two or three dorsal roots, intraoperative maps suggest a broader population-level rostrocaudal distribution of each somatosensory projections to motor neurons, stretching over as much as the entirety of the cervical and lumbar cord for some muscles (London et al., 2022;McIntosh et al., 2023;Phillips & Park, 1991;Schirmer et al., 2011).

This discrepancy emphasizes the importance of methodologies for functionally mapping the segmental distribution of muscle innervation (which includes motor neurons and somatosensory projections to motor neurons), and thus captures the inherent variability in these projections along the rostrocaudal extent of the human spinal cord. We refer to this muscle innervation map as*projectome*(Rowald et al., 2022). While invasive methodologies can help delineate these personalized projectomes, their inherent burden limits their widespread use in clinical applications. Alternatively, functional magnetic resonance imaging (fMRI), widely deployed to probe brain activity using the blood oxygenation level dependent (BOLD) contrast—a slow proxy for neural activity based on neurovascular coupling (Hillman, 2014)—can offer a non-invasive approach. Extending these imaging techniques to the spinal cord is, however, challenging, due to its small size, and the artifacts arising from physiological noise (Hutton et al., 2011;Krüger & Glover, 2001;Triantafyllou et al., 2005) and magnetic field inhomogeneities (Jezzard & Clare, 1999). Developments in MRI acquisition and analysis methods have progressively improved our ability to tackle these limitations (Cohen-Adad et al., 2021;De Leener et al., 2017;Frässle et al., 2021;Kasper et al., 2017;Kinany et al., 2022).

Most studies have utilized spinal cord fMRI to map activity in the cervical cord, mainly during movements or stimulations of the upper limbs (Kinany et al., 2022;Landelle et al., 2021). Instead, the potential to acquire functional MRI signals of the lumbosacral spinal cord to assess lower limb innervation has been poorly explored. This paucity of studies may be explained by the additional difficulties imposed by the small dimensions of the lumbosacral spinal cord (Donaldson & Davis, 1903;Frostell et al., 2016;Hynes et al., 1993;Ko et al., 2004;Mendez et al., 2021;Nunès et al., 2023;Rowald et al., 2022), the anatomical variability between individuals (Khasawneh, 2019;Nunès et al., 2023;Ranger et al., 2008;Van Schoor et al., 2015), the weaker signals acquired in this region of the body, as well as the lack of dedicated tools to process these signals. Most studies examining lumbosacral spinal cord activity (Jia et al., 2019;Kornelsen & Stroman, 2004,^2007^;Moffitt et al., 2005;Stroman et al., 2004) have actually relied on a non-BOLD contrast mechanism, called signal enhancement from extravascular water protons (SEEP) (Stroman et al., 2001a,2001b). However, the reliability of this methodology has been debated (Bouwman et al., 2008;Jochimsen et al., 2005). To date, only four studies have explored BOLD activity in the lumbosacral spinal cord: two in the context of resting-state recordings (Combes et al., 2023;Ricchi et al., 2024), one investigating the impact of echo time (TE) on a unilateral lower limb voluntary task (Kündig et al., 2024), and another leveraging fMRI for pre-operative planning and personalized protocols design for spinal cord stimulation (Rowald et al., 2022). Although the latter study involved only three patients, it underscored the potential of individualized mapping of projectomes. Particularly noteworthy was the fMRI-derived projectome of one patient, which revealed a counterintuitive mapping; an inversion of flexor and extensor muscles of the ankle compared to expectations, a finding subsequently validated intra-operatively.

Here, we aim to build on these promising results and provide the first systematic characterization of BOLD-derived muscle-related activity in the human lumbosacral spinal cord. To this end, we present a tailored fMRI pipeline for the acquisition of BOLD signals from the lumbosacral spinal cord. Our approach integrates an adapted T2*-weighted reduced field-of-view gradient-echo echo-planar-imaging protocol, which previously enabled reliable detection of cervical activation patterns (Kinany et al., 2019,^2020^,^2022^,^2024^;Weber et al., 2016,^2020^), with high-resolution structural MRI acquisitions (Rowald et al., 2022) muscle-specific activity within the lumbosacral cord and the anatomical mapping of the different levels of the spinal cord in each participant. We tested these protocols in 12 healthy participants during three distinct task paradigms: active joint-specific movements, passive stretching of specific muscles, and tendon vibration applied to specific muscles to evoke proprioceptive signals. We applied these paradigms to extensor and flexor muscles of the ankle, knee and hip joints of the right leg, in order to identify activity patterns related to each muscle at both the group- and participant-level. Finally, we propose a framework for constructing and interpreting personalized projectomes maps based on structural tracing of spinal roots in high-resolution anatomical images and task-based fMRI acquisitions. Our results lay the foundation for the development of a lumbosacral spinal cord fMRI pipeline to capture individualized maps of muscle innervation patterns in the human lumbosacral spinal cord. This approach holds promise for facilitating personalized treatment planning of clinical interventions to treat neurological deficits.

## 2 Methods

### 2.1 Participants

A total of 12 healthy participants were enrolled in the study (age: 26.5 ± 2.0 years; 5 female; 7 male; ethics project ID 2019-02177; seeTable S8for details). The study took place at the MRI facility of the Human Neuroscience Platform of the Fondation Campus Biotech Geneva, in Geneva, Switzerland and had been approved by the Commission Cantonale d’Éthique de la Recherche Genève (CCER, Geneva, Switzerland, 2019-02177). Ten healthy volunteers took part in three experimental paradigms (active movements, passive movements and tendon vibration). One participant only performed the active and passive recordings, and one only underwent the tendon vibration recording.

### 2.2 Experimental protocols

**Fig. 1.**
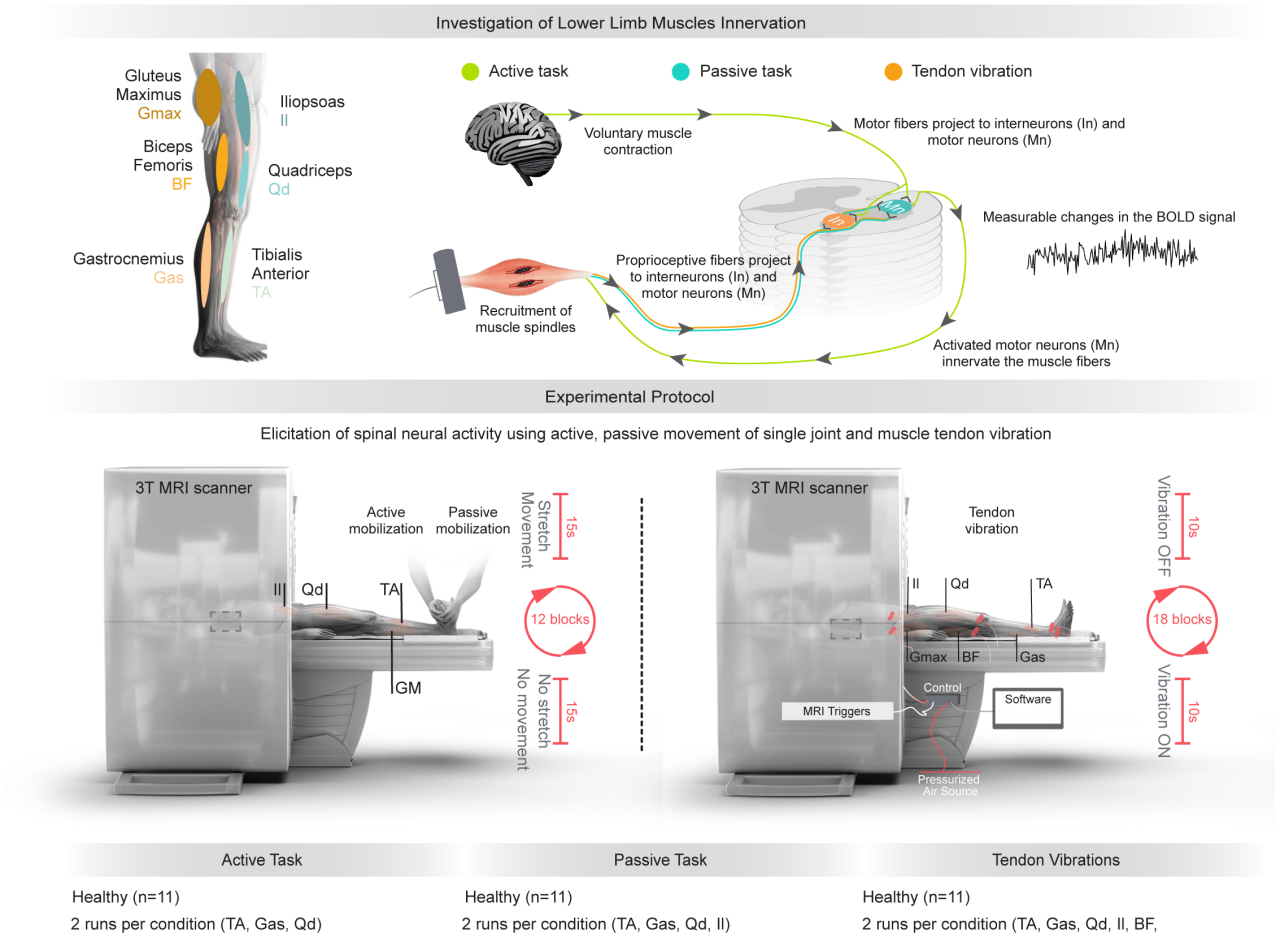
Experimental protocol for the three different conditions explored. Upper panel: Key muscles of the lower limb investigated in this study. The muscle spindles are recruited either by stretching the muscles in which they are embedded, or by applying muscle tendon vibration. Motor fibers are directly recruited through voluntary muscle contraction. Lower panel Left: During the ‘Active task’ condition, participants were asked to actively perform specific movements with their right leg: ankle extension (to activate Gas) and flexion (TA), knee extension (Qd). In the ‘Passive task’ condition, participants had their right leg moved by an experimentator trained by an experienced physiotherapist to passively execute specific movements: ankle extension (to activate muscle spindle endings in TA) and flexion (Gas), knee flexion (Qd), hip extension (Il). Lower panel Right: In the ‘Tendon vibration’ condition, participants underwent tendon vibrations using MR-compatible pneumatic vibrators (synchronized with MRI triggers) on extensor and flexor muscles of three lower limb joints of the right leg: ankle (TA/Gas), knee (Qd/BF) and hip (GMax/Il). Two runs are acquired for each muscle, for each condition.

In order to image the rostrocaudal activity of motor and somatosensory neuron pools linked to agonist / antagonist (extensor / flexor) muscles at the ankle (Gastrocnemius Medialis [Gas] / Tibialis Anterior [TA]), knee (Quadriceps [Qd] / Biceps Femoris [BF]) and hip (Gluteus maximus [Gmax] / Iliopsoas [Il]) levels, participants underwent four experimental sessions involving three different task paradigms: (i) Active movements, to recruit motor neurons by actively performing a specific movement, and (ii) Passive muscle stretch and (iii) Tendon vibrations, both used to recruit muscle spindle afferents, which innervate interneuron and motor neuron populations in the spinal cord (Ribot-Ciscar et al., 1998) (Fig. 1). For all conditions, tasks were performed with the right limb. Participants were positioned in the MRI scanner in supine position, and foam cushions were used to position the limbs comfortably and allow the different tasks to be performed.

#### 2.2.1 Active movements and passive muscle stretch

For the active and passive tasks of the ankle (TA/Gas) and knee (Qd) muscles, participants were positioned supine with supports under their knees and ankles, secured with a belt and sandbags to minimize movement. This setup allowed a researcher (trained by a certified physiotherapist) to effectively stretch the muscles and the participants to perform the active movements. For the passive task involving the stretch of the hip flexor (Il), participants were similarly positioned but with the upper body elevated on a hard support. Foam cushions were used for the head, elbows, hands, and the opposite knee and ankle, allowing the researcher to effectively stretch the hip flexor muscle (Il). Each joint was assessed in a different session. The knee and hip sessions were carried out on the same day. To not extenuate the participants, the ankle session was set on a different day. The order of the sessions (ankle, knee/hip) was randomized across participants. Two runs were acquired for each joint for both active and passive tasks, in a randomized order.

Audio cues were given to either the participant (active task) or the researcher (passive task). The movements were set in 12 blocks of 15 s of duration with 8 movement repetitions in each block, alternating with 13 rest periods of 15 s, making one run 6 min and 23 s (153 volumes). A high pitch audio cue indicated the beginning of a block. A low pitch audio cue indicated the beginning of each movement within the block.

For the active task, participants were asked to actively perform dynamic repetitions of specific movements with their right leg. The movements were shown to them before each acquisition. They were instructed to perform the movements with a partial yet large amplitude, maintaining controlled movements, and avoiding overstretching the antagonist muscle. The participants performed the movements following the audio cues (low pitch, movement duration of approximately 1.5 s). They practiced the movements before the acquisition until feeling comfortable performing them independently. Due to the physical constraints of moving in the MRI scanner, as well as to minimize the presence of significant motion artifacts, we limited the active movements to ankle extension (Gas) / flexion (TA) and knee extension (Qd). A researcher (trained by a certified physiotherapist) made sure to provide support to get the limb in resting position after each movement.

For the passive task, a researcher (trained by a certified physiotherapist) performed specific movements on the participant’s right leg in order to stretch the targeted muscles. Passive movements included ankle extension (TA) / flexion (Gas), knee flexion (Qd), and hip extension (Il).

#### 2.2.2 Muscle tendon vibration

MRI-compatible pneumatic vibrators were used to stimulate muscle spindle afferents (Kavounoudias et al., 2008;Landelle et al., 2020). Small amplitude (0.5 mm peak to peak) and constant frequency (70 Hz) vibrations were delivered using an SMC ITV2050 air-pressure regulator driving the rotation of eccentric ceramic spherical masses embedded in the vibrator turbine. The stimulation parameters were selected on the basis that small amplitude vibration activates preferentially primary muscle spindle endings, with responses linearly proportional to the vibration frequency up to 70–80 Hz (Roll & Vedel, 1982). A custom software implemented in the LabVIEW environment (National Instruments) allowed to synchronize the vibratory stimulation with the MRI acquisitions. This device did not produce artifacts in the fMRI scans, nor did it modify the signal-to-noise ratio, as already reported in previous studies during which vibrations were applied during brain fMRI acquisitions (Kavounoudias et al., 2008;Landelle et al., 2020).

Six pneumatic vibrators were attached to the participant’s right leg using elastic bands on the tendons of each pair of agonist/antagonist (extensor/flexor) muscles at the ankle (Gas/TA), knee (Qd/BF) and hip (GMax/Il) levels. For each joint, the protocol was divided into two runs for each pair of agonist/antagonist muscles. In each run, two vibrators, one located on the flexor muscle and the other on the extensor muscle, were alternatively activated in blocks of 10 s. The flexor muscle tendon is at rest when the extensor muscle tendon vibration is activated and vice-versa. Between tendon vibration blocks, periods between 10 and 15 s of delay were set. These interstimulus jitter timings were randomly set and recorded. One run consisted of 18 alternate vibration blocks, for a total of 6 min and 50 s (164 volumes). To avoid any bias, the order of the six runs was randomized. Prior to data acquisition, the participant, in a seated position, was first exposed to several preliminary vibrations to familiarize themselves with the sensation produced by the vibrators. They were asked to relax and to report any illusion of movement, thus attesting the correct placement of the vibrator by the activation of muscle proprioceptive afferents.

### 2.3 fMRI data acquisition

All participants were comfortably installed in a Prisma 3 Tesla scanner (Siemens Healthineers, Erlangen, Germany) in supine position. Spine (32-channel array) and body coils (16-channel array) (Siemens Healthineers, Erlangen, Germany) were used. The participants were instructed to relax, to remain still, and to breathe normally.

For every participant, one high-definition structural 3D axial T2-weighted SPACE (Sampling Perfection with Application-optimized Contrasts using different flip angle Evolution) with ZOOMit (dynamic excitation pulses to achieve selective/zoomed field-of-view) imaging (repetition time, TR = 2500 ms; echo time, TE = 106 ms; interpolated voxel size = 0.3 × 0.3 × 0.5 mm^3^; duration, 12 min) was acquired with the purpose of determining the participant-wise rostrocaudal distribution of lumbosacral spinal levels (L1-S2). A session-specific T2-weighted anatomical image (SPACE sequence with a resolution of 0.4 × 0.4 × 0.8 mm^3^; TR = 1500 ms; TE = 135 ms; duration, 6 min), extending from T9 to L4 vertebrae, was also acquired for registration and normalization purposes. Functional acquisitions were performed using a gradient-echo echo-planar sequence with ZOOMit field-of-view imaging (TR = 2500 ms; TE = 34 ms; FOV = 48 x 144 mm^2^; flip angle = 80°, in-plane resolution = 1.1 × 1.1 mm^2^; slice thickness = 3 mm). Note that, compared to cervical recordings (Kinany et al., 2019,^2020^,^2022^;Weber et al., 2016,^2020^), in-plane voxel size was increased to account for the additional volume of tissue in the lumbar region (*i.e.*, at the levels of the hips) and thus avoid folding artifacts. Manual shimming adjustments focused on the spinal cord were performed to adjust field homogeneity. Thirty-two axial slices were acquired per volume. At the start of every session, a localizer sequence where the vertebrae can be identified is acquired. The ankle joint session lasted approximately 90 min, while the knee and hip sessions were about 45 min each. The tendon vibration session lasted approximately 60 min.

### 2.4 Personalized spinal levels identification

We identified personalized lumbosacral spinal levels L1 to S2 (Fig. S2) using the high-resolution structural MRI (T2-weighted SPACE with ZOOMit imaging). First, rostro-caudal region of the L1 vertebrae, we identified the L1 ventral and dorsal roots entering the spinal canal and traced these roots in the rostral direction until their merging zone with the white matter. This merging zone defines the root entry zone (REZ) of L1 nerve roots. Once the L1 entry zone was identified, the other REZ were identified by moving rostrocaudally (T12, L2-S3). The spinal levels (L1-S2) were then delimited by the middle points rostrocaudally between the REZ (T12-S3).

### 2.5 fMRI data preprocessing

The fMRI preprocessing was carried out as previously described in (Rowald et al., 2022) using the FMRIB Software Library (FSL) v5.0 (Jenkinson et al., 2012) and the Spinal Cord Toolbox (SCT) v5.6 (De Leener et al., 2017).

A two-phase motion correction procedure was performed using FMRIB’s Linear Image Registration Tool (Jenkinson et al., 2002). First, the volumes of each run were averaged into a mean image. The centerline of the spinal cord was automatically extracted (Gros et al., 2018). A cylindrical mask of diameter 30 mm was drawn along it and used to exclude the regions outside the spinal cord. Within each run, all volumes were registered to the mean image using three-dimensional rigid body realignment (spline interpolation and least square cost function). To account for the non-rigid structure of the spinal cord, a two-dimensional slice-wise realignment (spline interpolation and least square cost function, with no z regularization) was then conducted, taking as reference the mean image of the corrected volume (Cohen-Adad et al., 2009). Motion parameters were retrieved, and the mean framewise displacement (FD) was computed for each run (averaged over slices and time). Finally, all runs corresponding to the same session in the scanner were aligned to the first run of the session using three-dimensional rigid body realignment (spline interpolation and least square cost function). All images were inspected. Any top or bottom slices not present in all runs were cropped out. Motion scrubbing was also performed with FSL’s tool to identify outliers volumes, using DVARS (the root mean square of the difference of intensity between consecutive volumes) metric in the spinal cord, with a box-plot cut-off (75th percentile + 1.5 x the interquartile range) (Power et al., 2014). Both the cerebrospinal fluid and the spinal cord were automatically segmented (with manual corrections when necessary) from the mean functional and the T2 anatomical images, using the SCT (Gros et al., 2019).

To eliminate non-neural confounds from the signals, such as those linked to physiological noise, we used component-based noise extraction (aCompCor (Behzadi et al., 2007)) through the PhysIO Toolbox (Kasper et al., 2017). The first five principal components of the CSF signal were extracted on the unsmoothed functional images and added as nuisance regressors.

The motion-corrected functional volumes were spatially smoothed, volume by volume, using a 3D Gaussian kernel (full width half maximum (FWHM) of 2 x 2 x 6 mm^3^) applied along the centerline of the spinal cord, to preserve consistency at the anatomical level.

Coregistration was performed within each participant (functional-to-anatomical). Using the SCT (De Leener et al., 2017), functional images were coregistered to the corresponding T2 anatomical image with multi-step non-rigid transformations, based first on the combined segmentations of CSF and spinal cord and then on the images themselves. Normalization warping fields (anatomical-to-PAM50 template) were also estimated. Importantly, standard vertebrae-based alignment commonly used for cervical images is not suited to normalize lumbosacral images, where the variability between spinal segmental levels and vertebral bodies is more important (Frostell et al., 2016). We proposed, instead, an alternative procedure using two other anatomical landmarks, the cauda equina and the lumbar enlargement (Fig. S2), which we labeled on the spinal cord segmentations of each anatomical image. The PAM50 template spinal cord segmentation was completed until the cauda equina and equivalent labels were put in place. The anatomical images were straightened along the spinal cord and non-linearly registered to the PAM50 template. The warping fields obtained for the coregistration (functional-to-anatomical) and normalization (anatomical-to-template) were concatenated to get the functional-to-template transformations.

### 2.6 fMRI data analysis

#### 2.6.1 Analyses in participant native space

Using the pre-processed functional volumes (motion corrected, smoothed) and the nuisance regressors (only physiological noise and motion outliers for the active and passive tasks, while motion parameters were also included for the tendon vibrations task), a first-level statistical analysis was performed for each individual run using FMRIB’s Improved Linear Model with local auto-correlation correction (Woolrich et al., 2001). Only runs where the mean FD was less than 0.5 mm were taken into account (seeTable S1). As explanatory variables, the timings of the task (block design) were convolved with three optimal basis functions using FMRIB’s Linear Optimal Basis Set (Woolrich et al., 2004), with the second and third waveforms orthogonal to the first waveform. The resulting parameter estimates for the two runs were passed through a fixed-effects model to obtain the second-level analysis (participant-, task-, and muscle-specific) activation maps. Voxels were considered significant at a Z-score > 2.3 (uncorrected). These results were then registered to the respective anatomical image to assess their spatial distribution with respect to participant-specific spinal levels (seeSection 2.7).

An additional third-level analysis (participant- and muscle-specific) was carried out to assess the global effect of all tasks for each muscle, in each participant. Only second-level results for which the mean FD was less than 0.5 mm for the two runs were taken into account (seeTable S1). The anatomical images from the different sessions were co-registered to the ankle session, thus defining a participant-specific common space in which all second-level results were warped. For every muscle, the different runs went through a fixed-effects model and third-level activation maps were obtained. Voxels were considered significant at a Z-score > 2.3 (uncorrected).

#### 2.6.2 Analyses in PAM50 template space

In order to allow for group-level analyses, second-level (participant-, task-, and muscle-specific) contrast of parameter estimates (COPE) and their variances (VARCOPE) were normalized to the PAM50 template. A fixed-effects model (group-, task-, and muscle-specific) was then carried out in a region extending from L1 to S2. Only second-level results for which the mean FD was less than 0.5 mm for the two runs were taken into account (seeTable S1). The voxel-wise threshold for active voxels was Z > 2.3 (corrected for multiple comparison using Gaussian Random Field (GRF) with a cluster-defining threshold of p < 0.05).

### 2.7 Spatial analysis

For both group- and participant-level activation maps, we monitored the number of active voxels in the different hemicords (anterior$n_{ventral}$, posterior$n_{dorsal}$, right$n_{right}$and left$n_{left}$). Dorsal-Ventral (DV) and Left-Right (LR) indices were computed:$LR_{index}=\frac{n_{left}-n_{right}}{n_{left}+n_{right}}$and$DV_{index}=\frac{n_{dorsal}-n_{ventral}}{n_{dorsal}+n_{ventral}}$(Seghier, 2008;Weber et al., 2016,^2020^). For the$DV_{index}$, a value of +1.0 indicates that all the active voxels are in the dorsal hemicord and a value of -1.0 indicates that all active voxels are in the ventral hemicord. Similarly, for the LR index, a value of +1.0 indicates that all the active voxels are in the left hemicord while a value of -1.0 indicates that all active voxels are in the right side.

We also computed the segmental distribution of the activity patterns at the group- and participant-level. Of note, the PAM50 template does not provide information on the segmental spinal levels below T12 (De Leener et al., 2018)^1^, thus lacking probabilistic maps of the lumbosacral region. To address this, we used the participant-specific rostrocaudal maps of the spinal levels from L1 to S2, defined as explained inSection 2.4, which were normalized to the PAM50 template to obtain probabilistic maps of the lumbosacral spinal levels from L1 to S2 for analyses in the template space (Fig. S2). These probabilistic levels were also adapted into non-overlapping contiguous binary maps (Fig. S2), so as to assess the rostrocaudal distributions of activity patterns in the template space. To this end, the normalized levels were first binarized (threshold of 0.6) and the different connected components computed (Silversmith, 2021), in order to keep only the largest one for each level. To ensure the continuity of the resulting component, we filled any holes in 3D. Components were dilated in 2D (x,y leaving z unchanged) to fill the entire in-plane extent of the spinal cord. If two levels were overlapping, the overlap region was divided equally (in the z-axis) between the levels. Similarly, if two levels were separated in the z axis, the void was filled and divided equally between the two adjacent levels. To monitor the laterality of activity patterns, these levels were combined to make a L1 to S2 continuous binary mask, which was subsequently divided into four hemicords.

For the analyses conducted in the native space, the participant-specific maps of spinal levels were used. We analyzed the rostro-caudal segmental distribution by examining the extent of active voxels (Z > 2.3) of the uncorrected second-level analysis (participant-, task-, and muscle-specific) and third-level analysis (participant- and muscle-specific) activation maps. A spinal level was labeled ‘active’ if it contained over 30% of the significant voxels. To adjust for the varying volumes of spinal segments, a size-correction factor was applied. This involved calculating the voxel count for each segment in each participant’s imaging session and normalizing these counts to obtain a correction factor representing their relative volume difference. Each number of active voxels was then divided by this correction factor, thus preventing rostro-caudal distributions to be driven by segment volume. The corrected counts of active levels were summed across all participants to form a histogram-like representation of activity distribution along the spinal cord for each task. This histogram was further normalized by its sum to fall within a range of 0 to 1, facilitating data comparison and interpretation.

### 2.8 Personalized projectomes

In order to better highlight and interpret the activation patterns obtained in each participant, we introduced a decision tree (Fig. S1) to construct personalized maps of the segmental distribution of muscle innervation (*i.e.*, personalized projectome). Specifically, we leveraged the participant-specific second- and third-level results to automatically obtain the most likely activation patterns for each muscle. A set of corrected maps (corrected for multiple comparison using GRF) was created with a threshold for active voxels at Z > 2.3 and a cluster-defining threshold at p < 0.05. The uncorrected map was added to the set, with a voxel-wise threshold for active voxels at Z > 2.3. The set was then iterated through with the following logic: (i) Maps from third level were given priority over those from second level; (ii) maps with a stricter Z threshold and cluster-defining threshold were given priority. We computed the different connected components (Silversmith, 2021) for each map. The components go through the decision tree and if their center of gravity is found within the reported range by the literature, it is kept as a candidate for the projection location of the muscle into the spinal cord. Its spatial location is then analyzed to determine if it corresponds to a motor neuron population pool or a sensory neuron population pool. We defined a component as interpretable with a high confidence component if it originates from a third-level corrected map. Note that we employed a similar procedure in a previous study (Rowald et al., 2022), where the personalized distribution of proprioceptive projections was obtained on three SCI patients and afterwards validated with intraoperative stimulations.

## 3 Results

### 3.1 Data quality assessment

Of a total of 220 runs, 62 were excluded due to excessive motion (*i.e.*, mean FD > 0.5 mm) (seeTable S1for details). The knee session for the active and passive tasks presented the most excluded runs (15 and 11, respectively). The ankle sessions for the active task presented the least excluded runs (5). In proportion fewer runs were excluded from the tendon vibration task (14) in comparison to the active (22) and the passive (23) tasks. One entire participant was excluded due to excessive motion in 18 out of 22 runs. Hence, not all participants or muscles underwent the participant-specific third-level activity patterns (muscle-specific, for combined tasks) analysis. When combining the three conditions (Active, Passive, Vibrations), participant-specific activity patterns for Gas and TA were obtained for 5 participants, and for Qd in 2 participants. Combining data from the Active and Vibrations conditions yielded Gas and TA patterns for 7 participants, and Qd patterns for 2. In the case of combining Passive and Vibrations conditions, Gas and TA patterns were obtained for 5 participants, Qd patterns for 4, and Il patterns for 6. Finally, merging data from the Active and Passive tasks provided Gas and TA patterns for 7 participants, and Qd patterns for 3. As anticipated, the ankle-involved tasks were more suitable for third-level analysis due to lesser movement in active and passive conditions, reducing the risk of exclusion associated with elevated FD.

The temporal signal-to-noise ratio (tSNR) gradually increased with the processing level: 5.72 ± 0.79 for raw data (mean over the spinal cord ± standard deviation, in the 158 runs kept in the analyses), 7.42 ± 1.03 after motion correction and realignment, and 23.27 ± 3.97 after smoothing (seeTable S2for details). The quality of the normalization was assessed. Preprocessed, non-excluded runs and their tSNR maps were normalized to the PAM50 template. The average mean functional image, and average tSNR across participants were then computed and visually inspected (Fig. S3).

### 3.2 Group-level activity

**Fig. 2.**
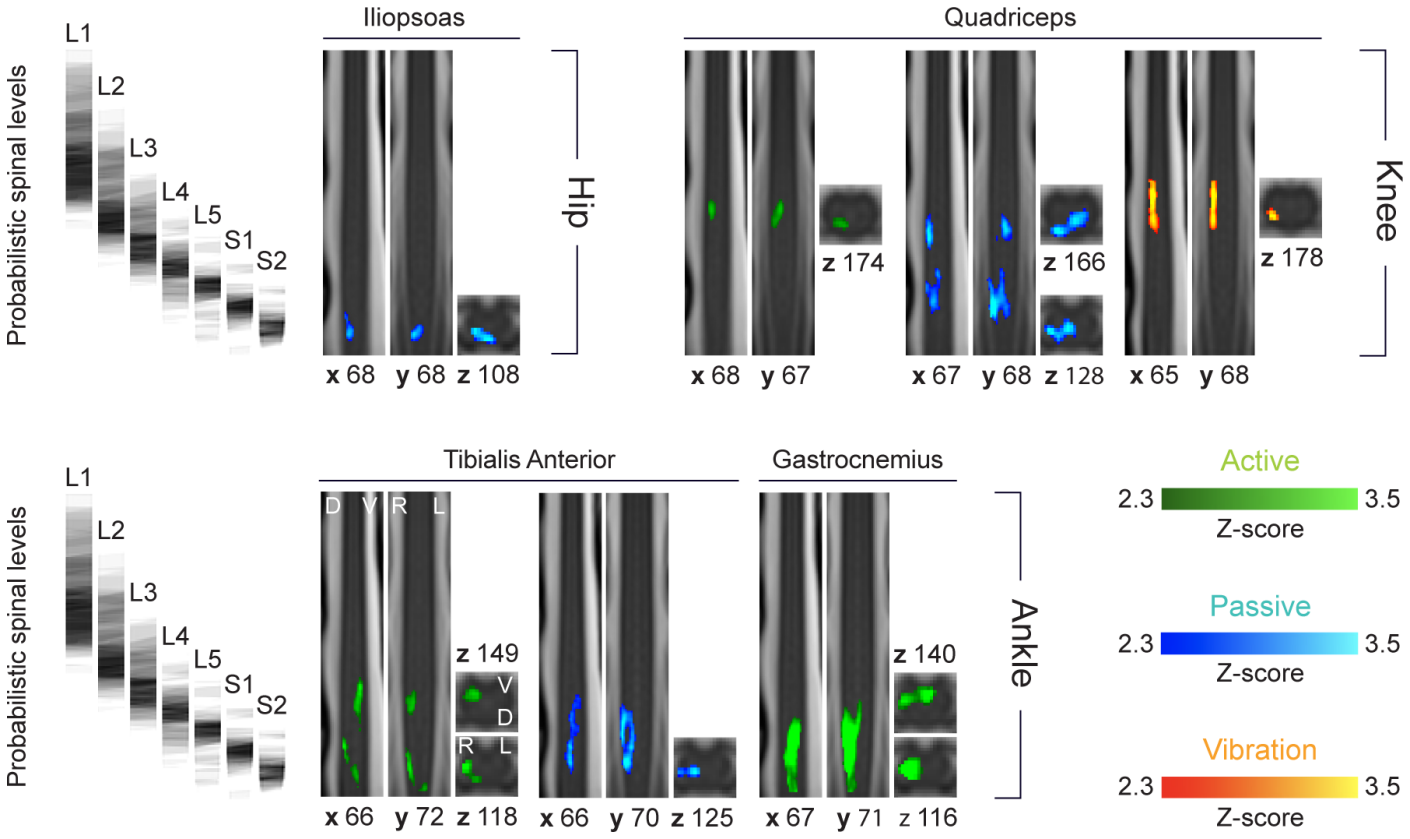
Group-level activity for all conditions and muscles. Group activation maps (fixed-effects modeling) for the different muscles of the hip (top row, left), knee (top row, right), and ankle (bottom row) joints for the different conditions (green for active movements, blue for passive ones, and orange for tendon vibration). Only the region extending from L1 to S2 was considered in the analyses. All maps are voxel-wise thresholded at a Z-score > 2.3, with a cluster-level corrected threshold of p < 0.05 to account for multiple comparisons. Only muscles for which significant activations were detected are illustrated (*i.e.*, maps for non-significant conditions are not shown). For each condition, a sagittal (left) and coronal (middle) views are presented, with their corresponding x and y position in the PAM50 space. Axial views and their corresponding z positions are provided on the right to highlight the in-plane distribution of activity. All maps are overlaid on the PAM50 template (De Leener et al., 2018). At the left of each row, we show normalized probabilistic spinal levels computed in this population.

Group-level activation maps were obtained for each muscle and task (Fig. 2). During the active task, all three muscles tested showed significant activations (Gas, TA, and Qd). In contrast, during the passive task, significant activation was observed in three out of the four muscles tested (TA, Qd, and Il), while no activity was detected in Gas. Regarding the tendon vibration task, significant activity was exclusively identified for Qd, with no significant activity reported for Gas, TA, BF, GMax, and Il.

To assess the lateralization of group-level activation maps, we examined the values of the left-right (LR) and dorso-ventral (DV) indices, which are summarized inTable S3. Consistent with the unilateral nature of the task, all conditions exhibited a strong right-sided lateralization, ipsilateral to the task, as illustrated by LR indices ranging from -0.48 (Il, Passive) to -1 (Qd, Active; TA, Passive; Qd, Vibrations). In the active condition, two muscles showed strongly negative DV indices (TA = -0.67, Gas = -0.74), indicating ventral activations. In contrast, the activation linked to Quad exhibited a marked dorsal preference (0.93). For passive movements and vibrations, nearly all activations were predominantly located dorsally (Passive: Quad = 0.19, Il = 0.83) and Vibration: Quad = 0.69). One passive activation showed a more ventral preference (Passive: TA = -0.74).

The rostrocaudal distribution of the activation patterns was evaluated and compared to the innervation maps derived from intraoperative nerve root stimulation studies (London et al., 2022;Phillips & Park, 1991;Schirmer et al., 2011). According to these studies, TA activations were expected between L4 to S1. During the active condition, the imaged patterns appeared more spread, with 43% of voxels in L3-L4 and 52% falling in S1-S2. In contrast, passive TA activation showed a very good alignment with the expected distribution, with 84% of the voxels found between L4 and S1. Regarding Gas, nerve root stimulation studies suggested innervation spanning L4 to S2. Accordingly, 91% of the activity related to Gas during active movements was localized in a region spanning L4 to S2 spinal segmental levels. For Quad activity, the expected distribution was from L1 to L4. This was largely true for the active (96% of the voxels in L2) and vibration (100% of the voxels between L1 and L2) conditions. However, passive movements elicited two main clusters of activity: one spanning L2 and L3 (42%) and one in L5 and S1 (49%). Finally, while Il is supposed to be innervated by segments L1 to L4, the observed activations were solely caudal, with all voxels falling in S1-S2 (seeTable S4for details).

### 3.3 Participant-level activity

**Fig. 3.**
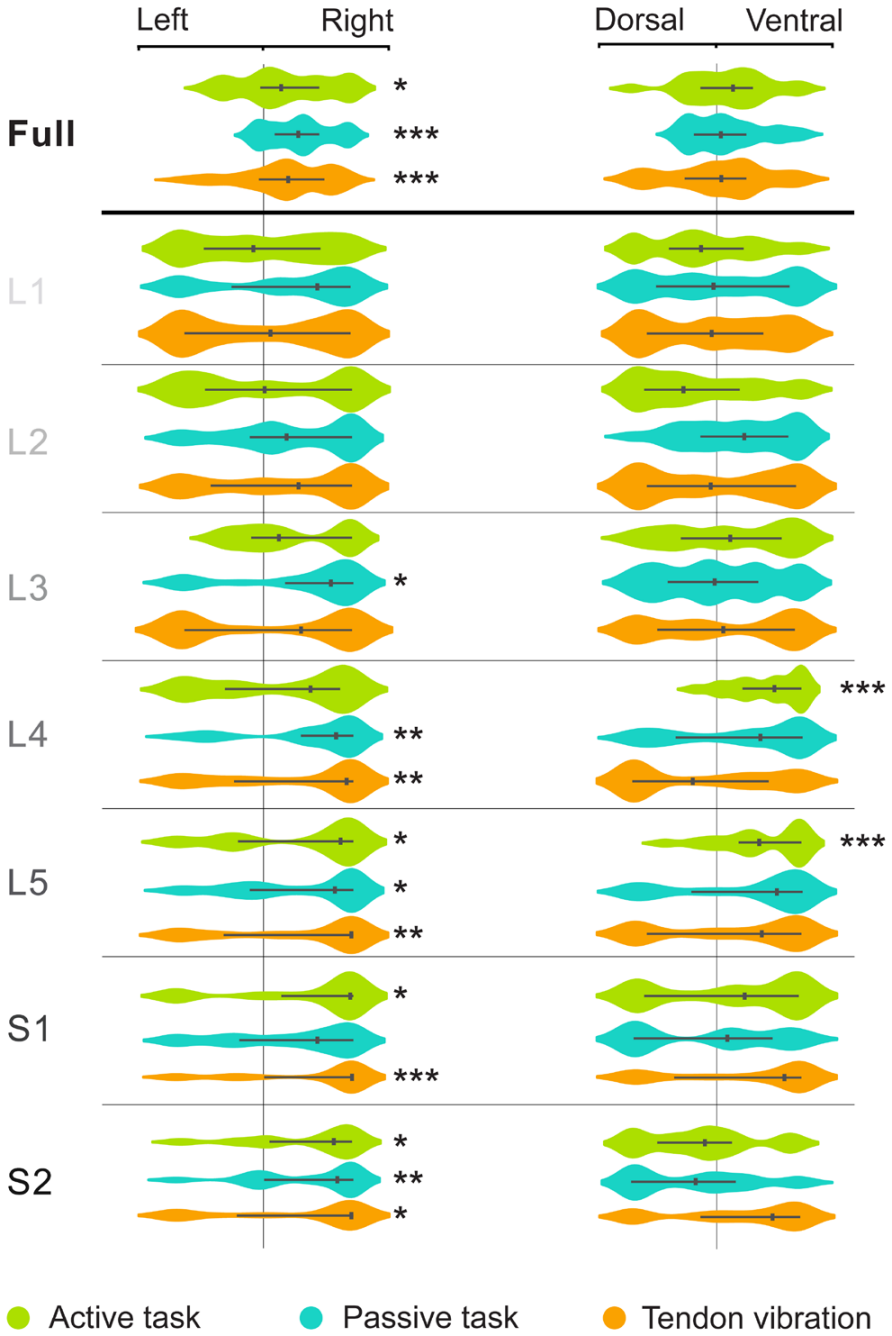
Lateralization of the participant-level activity patterns for the three conditions. Left-right (left panel) and dorso-ventral (right panel) laterality indices (from -1 to 1) for all three tested conditions, for the full spinal cord and independently for each spinal level (L1 to S2). Indices are computed using participant-specific second-level analysis (*i.e.,*across runs) (Z-score > 2.3, uncorrected). One-sided t-test results were corrected for false discovery rate (FDR) (Benjamini-Hochberg for 0.05 FDR). *p < 0.05, **p < 0.01, ***p < 0.001.

To characterize participant-specific second-level (*i.e.*, muscle- and task-specific) activity patterns, we first evaluated their left-right and dorso-ventral lateralization (Fig. 3,Table S5). Similarly, to group-level results, we observed a lateralization toward the ipsilateral right hemicord across all three conditions. This was visible when considering the ‘full’region (*i.e.*, L1 to S2 segments together) (one-sided t-test, p < 0.05 for the active task and p < 0.001 for the passive and tendon vibration tasks), as well as for most spinal levels and conditions, independently. We computed the mean LR index with its standard error for each task. The passive task showed the most lateralization with a negative value of -0.44 ± 0.07, followed by the vibration and active tasks with -0.29 ± 0.07 and -0.28 ± 0.11 respectively. Specifically, activations were significantly more present in the right hemicord for the active task in L5, S1, and S2, for the passive task in L3, L4, L5, and S2, and for vibrations in L4, L5, S1, and S2. No dorso-ventral lateralization was evident when considering the ‘full’ region. The active task had a mean DV index of -0.12 ± 0.10. The passive task also had a mean DV index of -0.12 ± 0.08. The vibrations task activations were even more central with a mean DV index of -0.02 ± 0.08. Nonetheless, significant ventral activity was detected at L4 and L5 for the active task.

We then conducted an evaluation of the participant-specific third-level activity patterns (muscle-specific, for combined tasks). For these third-level activation patterns, significant right hemicord lateralization was observed for the three conditions combined (one-sided t-test, p < 0.01), the active and passive combination (one-sided t-test, p < 0.001), and the passive and vibrations combination (one-sided t-test, p < 0.05). Additionally, significant ventral side lateralization was observed for these same combinations (one-sided t-test, p < 0.01).Table S5summarizes these findings.

**Fig. 4.**
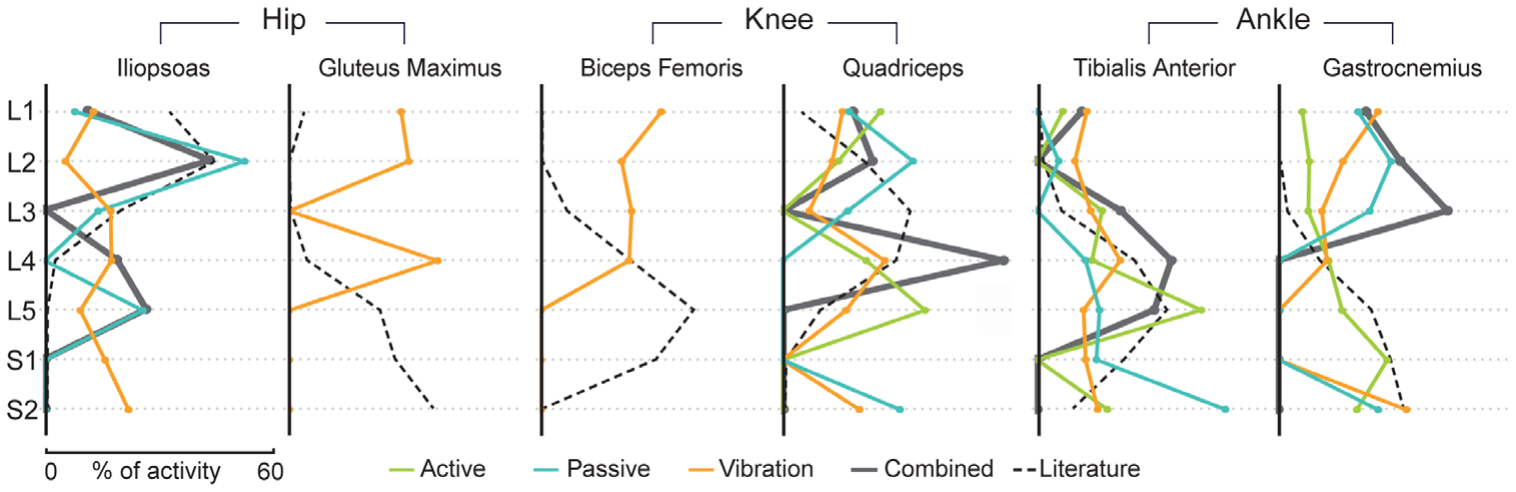
Rostrocaudal distribution of the participant-level activity patterns for the each muscle and condition. Distributions are computed using participant-specific second-level (*i.e.*, across runs, in blue [passive], green [active], and orange [vibration]) (Z-score > 2.3, uncorrected) and third-level (i.e, across conditions, in gray [combined]). Combined distributions correspond to the third-level active+passive+vibrations for TA, Gas, and Qd, and passive+vibration for Il. Activity percentage is calculated for levels L1 to S2, defined using participant-level high-resolution anatomical images, for each condition and muscle. Distributions are reported as normalized histograms of active spinal levels across participants. For a level to be counted as active, 30% of the participant-specific second-level (Z-score > 2.3, uncorrected) pattern must be within the spinal level. To adjust for the varying volumes of spinal segments, a size-correction factor was applied. Profiles derived from intraoperative nerve root stimulation are displayed for reference (London et al., 2022;Phillips & Park, 1991;Schirmer et al., 2011).

We proceeded to evaluate the activity patterns along the rostrocaudal direction, by analyzing their segmental distribution across spinal levels L1 to S2 (Fig. 4,Table S6). We observed that the activity patterns were widely distributed across spinal levels, and exhibited distinct profiles depending on the different conditions. Notably, while the overall profiles did not always align with those derived from intraoperative nerve root stimulation studies (London et al., 2022;Phillips & Park, 1991;Schirmer et al., 2011), we found that our results for TA largely concurred with these prior findings on somatosensory neuron projections to motor neuron pools. In particular, the combined activity profile displayed a peak around L4-L5 (66% of the activity in these two segments), consistent with the expected L4-L5 pattern (60%). Similarly, across the three conditions independently, a comparable caudal pattern was observed, with an average of 40% of the activity localized in the L4-L5 segments. Regarding Qd, combining conditions led to a peak in L4 (58% of activity), within the anticipated range of innervation. Although individual patterns exhibited significant rostrocaudal variations, the majority of their voxels were concentrated between L2 and L5 (74% for active, 52% for passive, 64% for vibration). In contrast, patterns linked to Gas activation for the different conditions were more heterogeneous, with only the active condition eliciting an activation in the expected L5-S2 range (65% of the activity). Likewise, ll activation led to a rostral profile (*i.e.*, 55% of activity observed above L4) that closely matched the anticipated patterns for the passive condition (74%), but not the vibration one (35%). For BF and GMax, exclusively examined using tendon vibration, both activation profiles were in disagreement with findings from intraoperative studies (London et al., 2022;Phillips & Park, 1991;Schirmer et al., 2011). In these cases, activity was predominantly located above L5, whereas literature suggests that these muscles are primarily innervated by caudal levels (L5-S1-S2) (seeTable S6for details).

### 3.4 Estimation of personalized projectomes

**Fig. 5.**
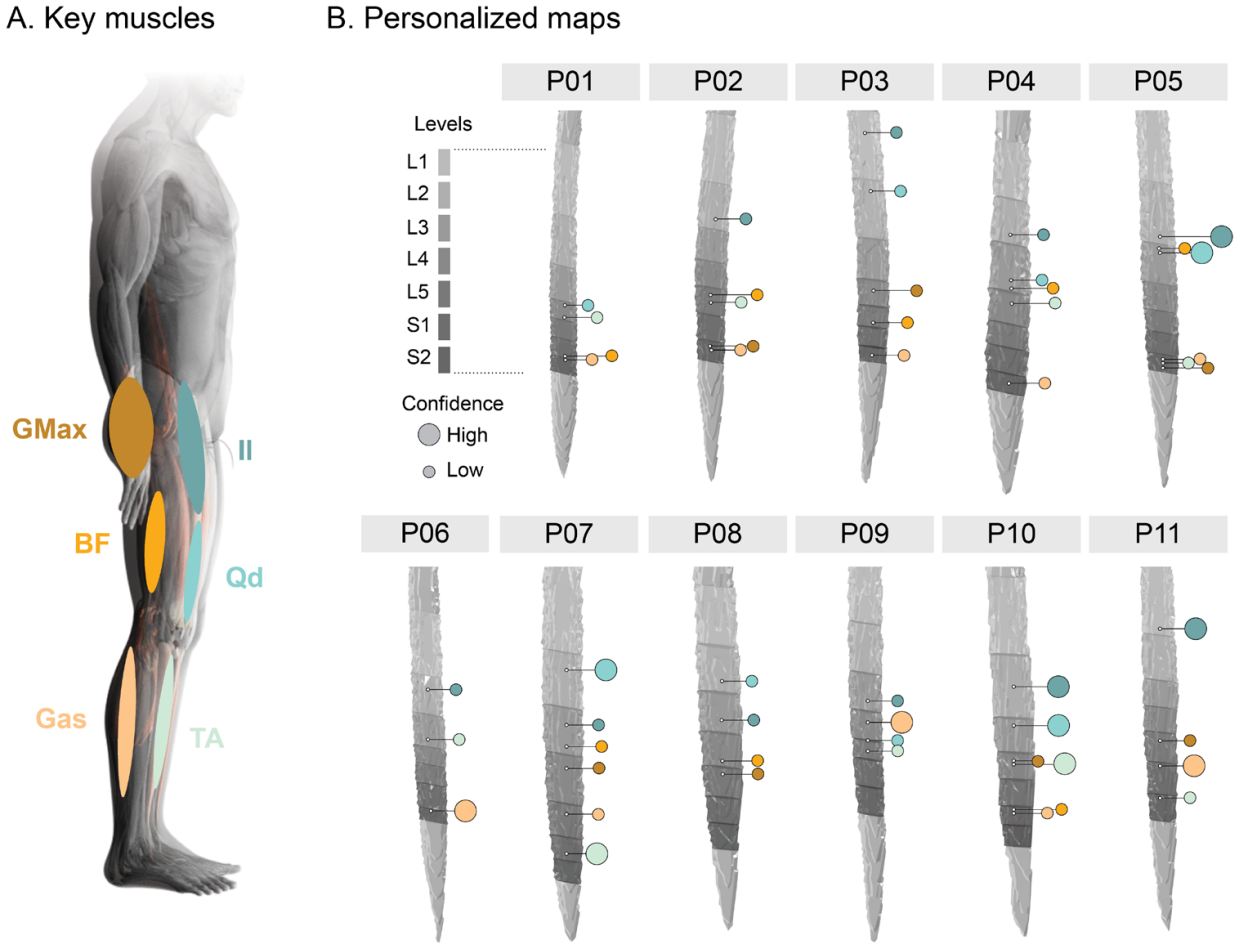
Personalized projectomes. (A) Key muscles included in the personalized innervation maps. Colors correspond to those used in panel B. (B) Personalized maps for 11 participants, built using the decision tree introduced inFigure S1. 3D models of the spinal cord, obtained from their T2-weighted anatomical scans, are presented for each participant. Participant-specific spinal cord segments, from L1 to S2, are overlaid using different shades of gray. Color circles indicate the likely innervation of the different muscles of interest. A large circle corresponds to a high level of confidence (component from participant-specific third-level multiple comparisons corrected activation pattern).

We built personalized projectomes for each participant (Fig. 5) using the decision tree introduced inFigure S1. In total, we were able to localize individualized innervation patterns for 52 muscles out of 64 tested. Among these muscles, 29 were derived from participant-specific third-level analysis (18 uncorrected, 11 corrected), and 23 from second-level analysis (22 uncorrected, 1 corrected). Most of the activity patterns selected were ipsilateral to the task, with 43 muscle innervations localized through activity in the right hemicord.

The innervation patterns of Il and Gas could be estimated in 10 participants (100% of Il between L1-L3 with 50% in L2; 80% of Gas in S1-S2 with 60% in S2). TA was reported for 9 participants (55% of TA in L4-L5 with 33% in L4). Qd and BF were both identifiable in 8 participants (75% of Qd in L2-L3; 50% of BF in S1-S2), while GMax could only be seen in 7 participants (71% in L4-L5).Table S7summarizes these findings.

Out of 11 participants, we were able to estimate the innervation patterns for all tested muscles in 4 individuals (note that P09 only underwent the active and passive movement conditions). For 3 participants, the innervation pattern of one muscle could not be estimated. In 3 participants, we could not estimate the innervation patterns of two muscles. Lastly, for 1 participant, the innervation patterns of three muscles were not estimable.

## 4 Discussion

Throughout our analyses, we found strong evidence for lateralization of the activity to the ipsilateral hemicord at the group- and participant-level, across all conditions. This is consistent with previous studies conducted in the cervical spinal cord (Weber et al., 2016,^2020^), and lends credence to the task-related nature of the imaged activation patterns. Although to a lesser extent, it should be noted that we also observed activity in the left hemicord. The involvement of interneuronal networks within the spinal cord, known to modulate sensorimotor processes and interlimb coordination, may contribute to this spread of activity to the contralateral side (Landelle et al., 2021;Purves et al., 2018). Alternatively, it could also be attributed to participants compensating for the task by activating muscles of the limb opposite to the one that was stimulated. Future work should consider the integration of MRI-compatible electromyography (EMG) sensors to monitor muscle activity and confirm this hypothesis.

The dorso-ventral lateralization of activity yielded interesting findings. While group-level analyses indicated a predominant ventral (motor) preference for active movements and a dorsal (sensory) preference for passive movements and tendon vibration, participant-level analyses presented a more complex picture. Activity patterns were distributed across both the dorsal and ventral hemicords, regardless of the task. This could be attributed to the limited statistical power of individual analyses, as well as to the use of uncorrected statistical maps. However, it is also plausible that the observed patterns reflect the complex interconnections between neurons in the spinal cord on a personalized basis. Indeed, active movements involve sensory and proprioceptive feedback, particularly during dynamic movements, which may contribute to the mixed dorso-ventral activations (Purves et al., 2018). A participant overdoing a dynamic movement, even if instructed against, would stretch the antagonistic muscle, triggering its stretch reflex, generating activities in both dorsal and ventral hemicords. Similarly, ventral activity during passive tasks may be indicative of monosynaptic reflex motor neuron pools (Purves et al., 2018). When muscle spindles fire during muscle stretch, the sensory information travels through type Ia and II fibers, the dorsal root ganglia and enters the spinal cord by the posterior horn. The fibers directly project on the motor neuron pools of the stretched muscle, in the ventral hemicord in addition to a complex network of interneuron connections (Purves et al., 2018). Ventral activity could also be the result of an active resistance by the participant to the passively induced stretch, even if instructed to relax. As for tendon vibration, the stimulation of proprioception can elicit distributed activations, traveling through type Ia and II fibers from the muscle. These fibers can project to both motor neuron and interneuron populations in the ventral and dorsal hemicords, respectively (Purves et al., 2018). Dorsal activity during the tendon vibration task can also be explained by cutaneous activity elicited by the vibrations, as cutaneous afferents project into interneurons located in the dorsal horn (Purves et al., 2018).

Lumbosacral imaging poses a major challenge due to the lack of dedicated tools, as most available processing methods have primarily been developed for the cervical region (De Leener et al., 2017). In particular, given our study’s objective to determine the rostrocaudal location of muscle innervation, we encountered two significant obstacles. Firstly, a substantial hurdle arose from the absence of normalization procedures specific to this region of the spinal cord. Indeed, the unique anatomy of this region, characterized by variable spinal segment lengths and locations relative to vertebral bodies (Donaldson & Davis, 1903;Frostell et al., 2016;Hynes et al., 1993;Ko et al., 2004;Mendez et al., 2021;Nunès et al., 2023), rendered conventional normalization based on vertebral landmarks suboptimal. Secondly, the lack of available atlases for defining the spinal segmental levels further complicated the task. In order to overcome the first limitation, we developed an alternative normalization procedure that utilized anatomical landmarks within the spinal cord, namely the cauda equina and the lumbar enlargement. This approach allowed us to account for inter-participant variation in spinal cord size. To address the second limitation, we adopted a methodology similar to that employed to characterize spinal segmental levels in the cervical spinal cord (Cadotte et al., 2015). Specifically, we capitalized on high-resolution structural imaging to identify the location of nerve roots in each participant. By combining these spinal levels with our normalization procedure, we successfully generated the first probabilistic maps of lumbosacral spinal levels in the PAM50 template.

Crucially, the utilization of participant-specific spinal levels and their corresponding maps in the PAM50 space enabled us to elucidate the rostro-caudal location of task-related activation patterns. At the group-level, fMRI-derived muscle-specific rostro-caudal organization were partially in agreement with studies using invasive nerve root stimulation, depending on condition (London et al., 2022;Phillips & Park, 1991;Schirmer et al., 2011). Overall, active movement performed best at reproducing the group-level rostro-caudal organization. Passive movement performed second best, but differed significantly during iliopsoas activation, which appeared in the sacral segments (S1-S2) rather than the expected region of innervation (L1-L2). We hypothesized that this might indicate inadvertent contraction of the antagonistic muscle (GMax) during the experiment, which is assumed to be present in S1-S2. Tendon vibration performed the worst out of all conditions in reproducing group-level activities, except for quadriceps stimulation, which elicited activation in the expected spinal levels. We posit that the performance for this muscle may be related to ease of identifying its tendons due to their size, reducing variability coming from vibrator placement. Even though tendon vibration was tested pre-imaging in a seated position, participants had more difficulty in experiencing the illusions of movement once lying down, particularly those evoked by stimulation of the hip muscles. Moreover, it should be noted that our tendon vibration protocol alternated agonistic and antagonistic vibrations, without dedicated rest periods where neither tendon was activated. While this streamlined experimental time, it raises the possibility of residual stimulation from previous vibrations affecting subsequent ones (Duclos et al., 2007). Therefore, we suggest that future studies include exclusive rest periods blocks in their tendon vibration protocol to ensure a more accurate investigation. Tendon vibration provides access to a wider range of muscles, such as the GMax and the BF, compared to active and passive movement which are constrained by the limited access to specific muscles by the experimenter and the spatial freedom for movement within the MRI scanner. Further, while active and passive movements are susceptible to variability due to human execution, tendon vibrations may be standardized across setups, ensuring consistent quality of results. For these reasons, we endorse further research into tendon vibrations, despite it showing the least favorable performance in our experiments.

At the participant-level, results were more equivocal, with the rostrocaudal distribution of uncorrected maps presenting deviations from intra-operative measurements for several conditions and muscles. These discrepancies could be attributed to various factors, both physiological and methodological. From a physiological perspective, inter-participant variability in muscle innervation is indeed expected (London et al., 2022). In addition, previous studies have underlined the existence of monosynaptic heteronymous Ia excitation in the lower limb, specially reported for specific muscle groups (Gas-BF and Gas-Qd) (Marchand-Pauvert & Nielsen, 2002;Marque et al., 2001;Meunier et al., 1994;Pierrot-Deseilligny & Burke, 2012). These inter-segmental connections in the spinal cord could contribute to the spread of rostral-caudal activity. Note that in these studies only a limited set of muscles were explored and more inter-segmental projections could be in place. Lumbosacral cord fMRI could be a tool to further explore these co-activations in humans, in the context of large cohort studies. On the methodological front, it is noteworthy that eliciting muscle activations led to significant motion artifacts, leading to the exclusion of 62 out of 220 runs. As such, participant-level maps may be impacted by the limited amount of data available. To mitigate this issue, we recommend that future studies consider increasing the number of runs to compensate for any excluded data. Additionally, further efforts on improving spinal cord motion correction could be undertaken.

To enhance the extraction of information from participant-level acquisitions, we developed a framework for systematically generating personalized projectomes. These summarized activity maps offer several advantages, including the highlighting of inter-participant variability, facilitation of interpretation, and the incorporation of an assessment of confidence in the resulting patterns. Here, even though we were able to estimate the innervation patterns for at least 80% of tested muscles in 7 out of 11 participants, delineating reliable innervation maps for all muscles and participants proved challenging. Despite these challenges, personalized projectomes, possibly obtained using a larger volume of individual data, can increase the clinical potential of the proposed lumbosacral fMRI tools. Notably, personalized projectomes have already demonstrated their utility in optimizing the placement of targeted epidural electrical stimulation (EES) implants on the lumbosacral cord of spinal cord injury (SCI) patients, with the aim of restoring leg and trunk motor functions (Rowald et al., 2022). In the same vein, probing the evolution of personalized projectomes over time holds promise for studying plasticity in both healthy and clinical cohorts, potentially shedding light on the effects of neurological disorders on spinal cord organization.

Our study has several limitations that warrant acknowledgment. Firstly, the small number of participants may have limited the statistical power of certain results, and the use of fixed-effect analyses restricts the generalizability of our group-level findings beyond the study population. Additionally, the repeatability of the activity maps has not yet been evaluated—that is, we have not repeated the scan sessions to generate personalized projectomes from new datasets for the same individuals. However, even with this limited sample size, our dataset provides consistent and valuable insights into lumbosacral spinal cord activity across multiple conditions. The limited number of participants was also compounded by excessive motion during the imaging sessions, leading to the exclusion of a significant amount of data. In particular, executing active and passive movements of the proximal lower limbs, targeting specific muscles without inducing motion in the lumbosacral region was challenging. Improving participant preparation and stabilization, limiting movement amplitude, and ensuring they do not resist movement may partially mitigate this issue and enhance data quality across all tasks. The use of isometric contractions in place of dynamic movements may also be explored. Lastly, we recommend for future studies to optimize task paradigms, including parameters such as run length, the number of runs, block duration, and rest periods. By fine-tuning these aspects, researchers may be able to achieve more robust and reliable results at the participant-level.

Disentangling lumbosacral spinal cord activity remains a challenging and complex task. In this study, we have taken the first step, to our knowledge, toward systematically examining BOLD activity in the lumbosacral spinal cord across an extensive range of tasks, encompassing three conditions and six distinct lower limb muscles. Furthermore, the introduction of a systematic pipeline for constructing personalized projectomes through high-resolution MRI and task-based fMRI represents a novel approach with the potential to enhance lumbosacral spinal cord fMRI. We hope that these initial investigations will contribute to the advancement of the field of lumbosacral spinal cord fMRI and drive the development of robust diagnostic tools to disentangle the functional organization of the spinal cord for future clinical applications.

## Supplementary Material

Supplementary Material

## Data Availability

Data and code will be made available upon reasonable request to the corresponding author.

## Footnotes

1 As of SCT version v5.6. Spinal levels information was updated since SCT version 6.1.
