# Supplementary material for: Towards personalized mapping through lumbosacral spinal cord task fMRI
Source: Imaging Neurosci (Camb). 2025 Jan 23;3:imag_a_00455. doi: 10.1162/imag_a_00455 (PMC12319805; doi:10.1162/imag_a_00455)
Supplement: Supplementary Material [file imag_a_00455-supp.pdf]

Supplementary information

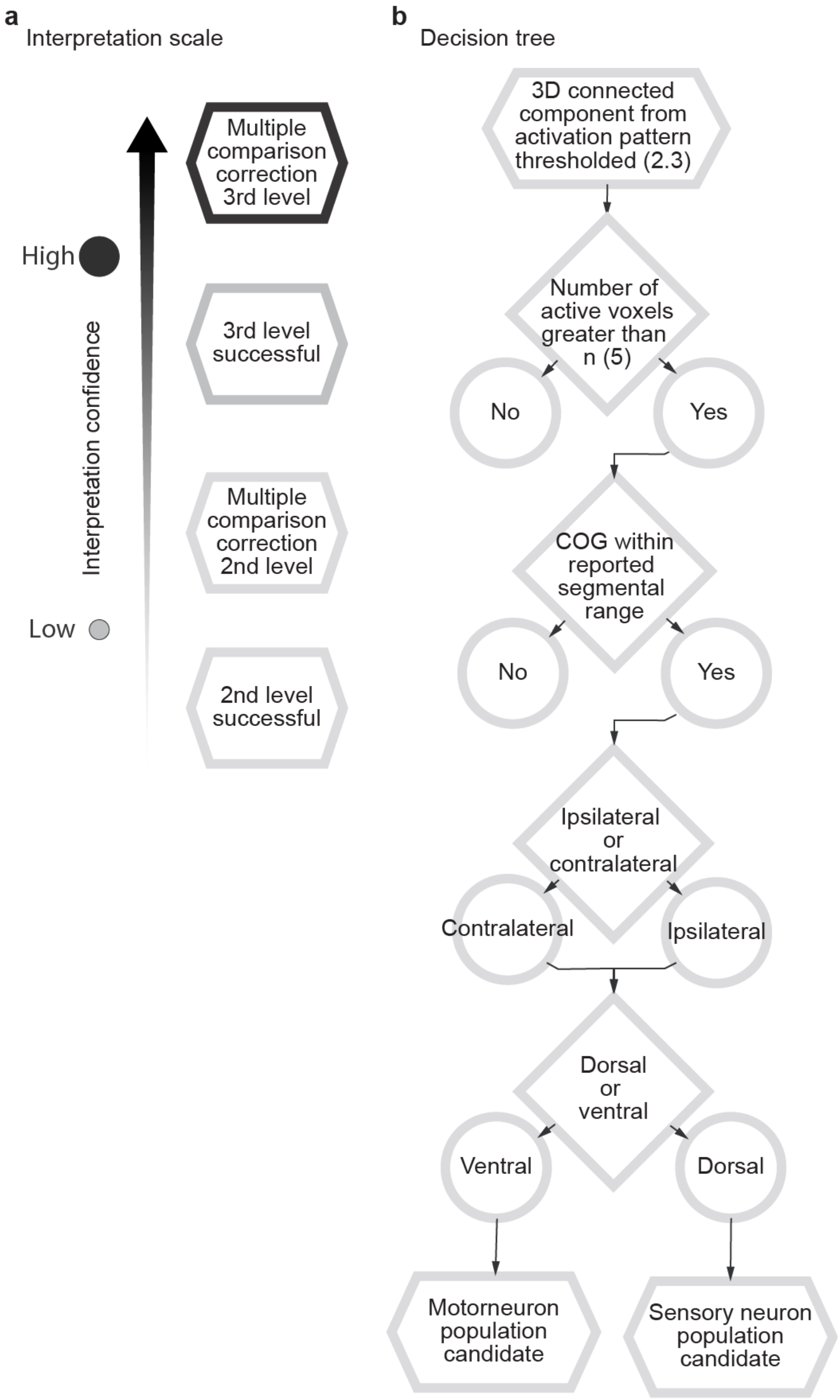

Figure S1. Decision tree for interpreting activity patterns to build personalized projectomes. Panel A/Step 1. Confidence in the interpretation of the activity pattern depends on the level of analysis from which it derives. **Panel B/Step 2.** Personalized projectome is built by following the decision tree to interpret the resulting activity patterns. 3D connected components are identified from thresholded ($Z > 2.3$) uncorrected or corrected participant-specific activity patterns. Each component goes through the decision tree. If the component has less than a certain number of active voxels (5) it is considered as noise and is discarded. Only components which are within the range reported from direct experimental measurements are kept. Motor neuron and sensory neuron population pool candidates are identified. Multiple comparison results (third level, or second level) increase the level of confidence of interpretation. Lateralizations in the anterior-posterior and left-right sides of the activity patterns are used to additionally identify homonymous projections. Activation patterns from a larger quantity of data have a greater confidence (3rd level gathers data from 4 or 6 runs, 2nd level gathers data from 2 runs). The components originated from participant-specific third-level activations with multiple comparison corrections have the highest interpretation confidence.

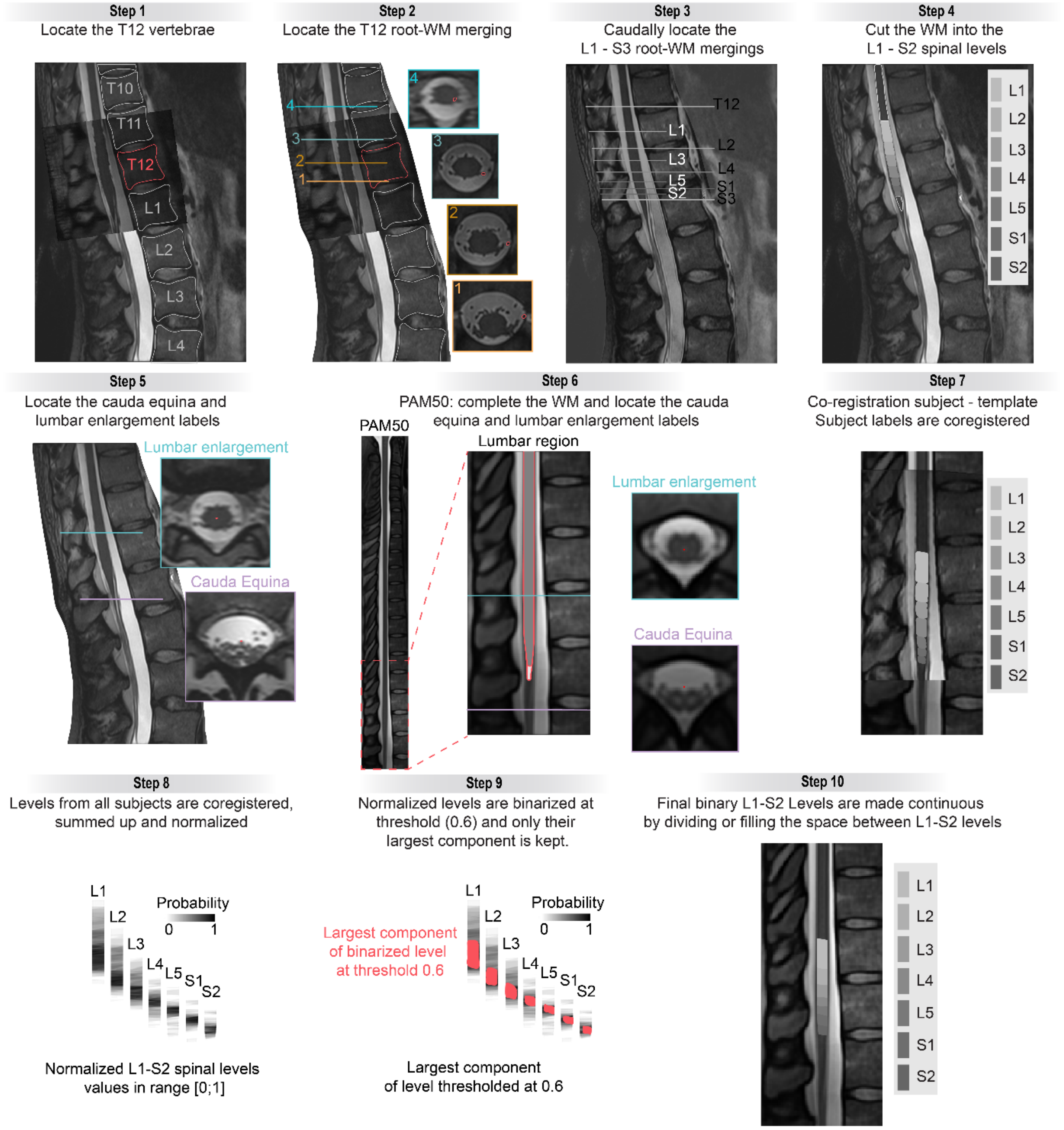

Step 1
Locate the T12 vertebrae
T10
T11
T12
L1
L2
L3
L4
Step 2
Locate the T12 root-WM merging
4
3
2
1
Step 3
Caudally locate the
L1 - S3 root-WM mergings
T12
L1
L2
L3
L4
L5
S1
S2
S3
Step 4
Cut the WM into the
L1 - S2 spinal levels
L1
L2
L3
L4
L5
S1
S2
Step 5
Locate the cauda equina and
lumbar enlargement labels
Lumbar enlargement
Cauda Equina
Step 6
PAM50: complete the WM and locate the cauda
equina and lumbar enlargement labels
PAM50
Lumbar region
Lumbar enlargement
Cauda Equina
Step 7
Co-registration subject - template
Subject labels are coregistered
L1
L2
L3
L4
L5
S1
S2
Step 8
Levels from all subjects are coregistered,
summed up and normalized
L1
L2
L3
L4
L5
S1
S2
Probability
0
1
Normalized L1-S2 spinal levels
values in range [0;1]
Step 9
Normalized levels are binarized at
threshold (0.6) and only their
largest component is kept.
Largest component
of binarized level
at threshold 0.6
L1
L2
L3
L4
L5
S1
S2
Probability
0
1
Largest component
of level thresholded at 0.6
Step 10
Final binary L1-S2 Levels are made continuous
by dividing or filling the space between L1-S2 levels
L1
L2
L3
L4
L5
S1
S2

Figure S2. Methodology to obtain the lumbosacral spinal levels (L1-S2) in the participant and PAM50 template spaces. Step 1. The high-resolution ZOOMit MRI volume is overlaid on the standard T2 anatomical MRI volume. Using a localizer image, the T12 vertebra is identified. Step 2. Starting from the most caudal slice of the identified T12 vertebra, the volumes are viewed axially and examined slice by slice in the rostral direction. The T12 spinal roots, (for instance, the T12 dorsal right root – marked in red), are identified as they enter (1) the spinal canal, approach (2,3) the spinal cord, and finally merge (4) with the white matter. **Step 3.** From the point where the T12 root merges with the white matter, an axial navigation slice by slice in the caudal direction is performed to identify where the roots from L1 to S3 merge with the white matter. **Step 4.** The spinal levels are determined by halving the rostrocaudal distance between roots. Using these T12-S3 root-white matter mergings, participant-specific L1-S2 spinal levels are defined. **Step 5.** The cauda equina and lumbar enlargement are identified, and two labels are placed at the center of the spinal canal at those slices. **Step 6.** The PAM50 cord segmentation is completed to include the tip of the sacral cord. As in Step 5, labels are placed at the center of the spinal canal at the cauda equina and lumbar enlargement slices. **Step 7.** Using these labels and spinal cord segmentations, the participant-specific anatomical image and spinal levels are co-registered with the PAM50 template. **Step 8.** All spinal levels from all participants are co-registered. At each spinal level, the participant-specific levels are summed up and normalized to produce probability maps for L1-S2. **Step 9.** The normalized levels are binarized with a threshold of 0.6, and only the largest connected component for each level is kept. **Step 10.** The space between the largest connected components of each level is either filled or divided to create a continuous, non-overlapping set of binary L1-S2 spinal levels in the PAM50 space.

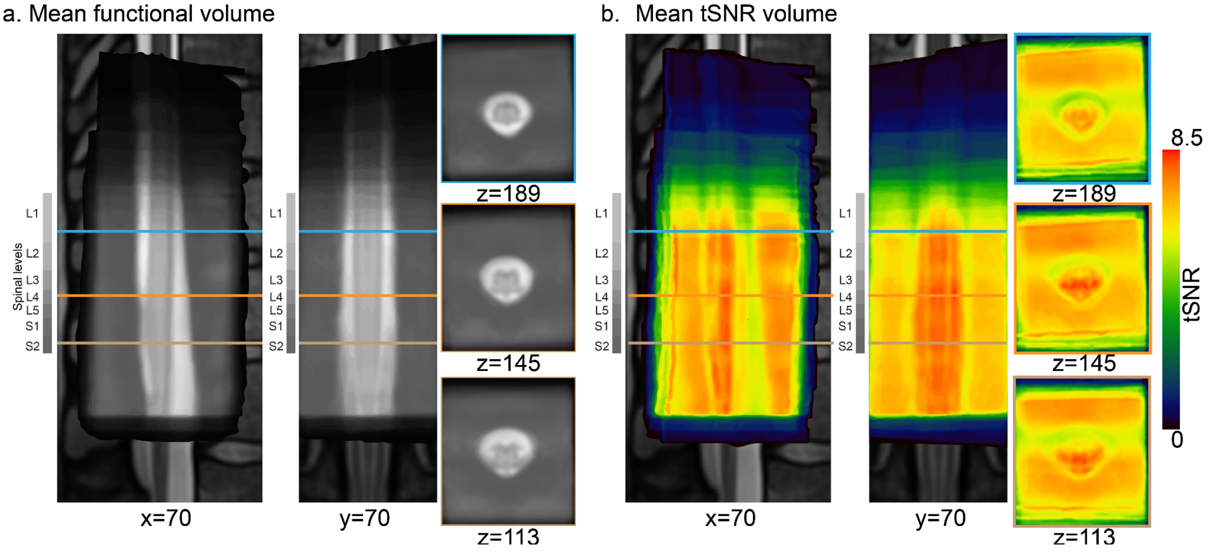

Figure S3. Mean functional and mean tSNR volumes in the PAM50 template space. a. Non-excluded runs were averaged after motion corrections and realignment and normalized to the PAM50 template. Within the area of interest (L1-S2 spinal levels) the CSF, WM and gray matter are clearly well defined. b. The tSNR of non-excluded runs, after motion correction and realignment were normalized to the PAM50 template and averaged. **Within the area of interest (L1-S2 spinal levels), the tSNR is higher in the WM and gray matter.**

Supplementary Table 1. Exclusions of runs due to motion.

Condition	Muscle	Total runs	Runs excluded (Mean FD > 0.5)
Active	Gas	22	2
Active	TA	22	5
Active	Qd	22	15
Passive	Gas	22	6
Passive	TA	22	6
Passive	Qd	22	11
Passive	Il	22	5
Vibrations	Gas and TA	22	5
Vibrations	Qd and BF	22	5
Vibrations	Il and Gmax	22	4
Total		220	64

Supplementary Table 2. tSNR for pre-processing stages

Stage	Raw acquisition	Motion corrected	Realigned	Smoothed
All runs (including motion excluded)	5.481 ± 0.826	6.556 ± 0.874	7.192 ± 1.074	21.691 ± 4.435

Runs after exclusion due to motion	5.716 ± 0.778	6.765 ± 0.842	7.424 ± 1.035	23.266 ± 3.966

Supplementary Table 3. Laterality spatial analysis for group level results in the PAM50 space

Condition	Muscle	Multiple comparison corrected	Voxels L	Voxels R	Voxels D	Voxels V	LR Index	DV Index
Active	Gas	Yes	300	1645	267	1818	-0.69	-0.74
Active	TA	Yes	133	742	145	742	-0.70	-0.67
Active	Qd	Yes	0	175	188	7	-1.00	0.93
Passive	TA	Yes	0	818	105	713	-1.00	-0.74
Passive	Qd	Yes	343	1102	940	637	-0.53	0.19
Passive	Il	Yes	63	179	251	23	-0.48	0.83
Vibrations	Qd	Yes	0	363	307	56	-1.00	0.69
Passive	Gas	No	47	362	37	396	-0.77	-0.83
Vibrations	Gas	No	5	31	25	11	-0.72	0.39
Vibrations	TA	No	3	32	29	8	-0.83	0.57
Vibrations	Il	No	171	153	51	290	0.06	-0.70
Vibrations	BF	No	92	127	51	172	-0.16	-0.54
Vibrations	GMax	No	39	118	123	75	-0.50	0.24

Supplementary Table 4. Rostrocaudal spatial analysis for group level results in the PAM50 space

Task			Spinal levels						
Condition	Muscle	Multiple comparison corrected	L1	L2	L3	L4	L5	S1	S2
Active	Gas	Yes	0	0	173	358	594	522	293
Active	TA	Yes	0	28	220	99	9	183	208
Active	Qd	Yes	8	187	0	0	0	0	0
Passive	TA	Yes	0	0	52	216	256	218	76
Passive	Qd	Yes	0	452	213	89	375	406	42
Passive	Il	Yes	0	0	0	0	0	40	229
Vibrations	Qd	Yes	135	228	0	0	0	0	0
Passive	Gas	No	0	124	54	0	2	104	147
Vibrations	Gas	No	3	9	14	3	7	0	0
Vibrations	TA	No	4	1	2	2	4	23	1
Vibrations	Il	No	2	7	39	1	2	85	68
Vibrations	BF	No	0	89	82	32	1	4	10
Vibrations	GMax	No	122	6	66	0	2	2	0

Supplementary Table 5. Laterality spatial analysis on the participant level results

Analysis	Condition (s)	Spinal Levels	Count	LR Index (mean ± se)	t-statistic	p-val (more right)	p-val (more left)	DV Index (mean ± se)	t-statistic	p-val (more ventral)	p-val (more dorsal)
Participant-Specific Second Level	Active	**All**	21	**-0.28 ± 0.11**	-2.01	**2.90E-02**	9.71E-01	-0.12 ± 0.10	-1.19	1.24E-01	8.76E-01
		L1	13	0.03 ± 0.21	0.17	5.65E-01	4.35E-01	0.17 ± 0.18	0.97	8.25E-01	1.75E-01
		L2	16	-0.04 ± 0.21	-0.21	4.18E-01	5.82E-01	0.23 ± 0.17	1.32	8.97E-01	1.03E-01
		L3	13	-0.3 ± 0.16	-1.80	4.82E-02	9.52E-01	-0.16 ± 0.19	-0.84	2.09E-01	7.91E-01
		L4	17	-0.21 ± 0.19	-1.08	1.49E-01	8.51E-01	**-0.59 ± 0.10**	-5.91	**1.09E-05**	1.00E+00
		**L5**	17	**-0.39 ± 0.19**	-2.12	**2.49E-02**	9.75E-01	**-0.51 ± 0.12**	-4.14	**3.88E-04**	1.00E+00
		**S1**	17	**-0.49 ± 0.19**	-2.60	**9.71E-03**	9.90E-01	-0.17 ±0.20	-0.84	2.07E-01	7.93E-01
		**S2**	12	**-0.5 ± 0.19**	-2.67	**1.10E-02**	9.89E-01	0.19 ± 0.19	1.03	8.38E-01	1.62E-01
	Passive	**All**	27	**-0.44 ± 0.07**	-5.62	**3.30E-06**	1.00E+00	-0.12 ± 0.08	-1.44	8.13E-02	9.19E-01
		L1	18	-0.26 ± 0.19	-1.37	9.42E-02	9.06E-01	-0.02 ± 0.19	-0.13	4.50E-01	5.50E-01
		L2	22	-0.28 ± 0.15	-1.92	3.40E-02	9.66E-01	-0.26 ± 0.13	-1.93	3.36E-02	9.66E-01
		**L3**	21	**-0.43 ± 0.16**	-2.69	**7.08E-03**	9.93E-01	-0.01 ± 0.15	-0.10	4.61E-01	5.39E-01
		**L4**	19	**-0.51 ± 0.16**	-3.26	**2.16E-03**	9.98E-01	-0.29 ± 0.18	-1.63	5.98E-02	9.40E-01
		**L5**	20	**-0.40 ± 0.16**	-2.52	**1.04E-02**	9.90E-01	-0.34 ± 0.18	-1.92	3.47E-02	9.65E-01
		S1	16	-0.32 ± 0.18	-1.74	5.15E-02	9.49E-01	0.11 ± 0.2	0.55	7.03E-01	2.97E-01
		**S2**	16	**-0.49 ± 0.16**	-3.15	**3.31E-03**	9.97E-01	0.28 ± 0.18	1.57	9.32E-01	6.85E-02
	Vibrations	**All**	50	**-0.29 ± 0.07**	-3.28	**9.50E-04**	9.99E-01	-0.02 ± 0.08	-0.29	3.88E-01	6.12E-01
		L1	38	-0.03 ± 0.14	-0.20	4.21E-01	5.79E-01	0.06 ± 0.12	0.48	6.84E-01	3.16E-01
		L2	36	-0.20 ± 0.14	-1.49	7.22E-02	9.28E-01	-0.01 ± 0.13	-0.06	4.76E-01	5.24E-01
		L3	34	-0.08 ± 0.16	-0.51	3.08E-01	6.92E-01	-0.10 ± 0.14	-0.70	2.44E-01	7.56E-01
		**L4**	34	**-0.35 ± 0.14**	-2.56	**7.67E-03**	9.92E-01	0.16 ± 0.14	1.13	8.67E-01	1.33E-01
		**L5**	29	**-0.38 ± 0.15**	-2.48	**9.61E-03**	9.90E-01	-0.17 ± 0.16	-1.10	1.41E-01	8.59E-01
		**S1**	25	**-0.54 ± 0.15**	-3.62	**6.92E-04**	9.99E-01	-0.32 ± 0.17	-1.92	3.34E-02	9.67E-01
		**S2**	23	**-0.38 ± 0.18**	-2.18	**2.00E-02**	9.80E-01	-0.27 ± 0.16	-1.64	5.74E-02	9.43E-01
Participant-Specific Third Level	Active & Passive & Vibrations	**All**	10	**-0.35 ± 0.12**	-2.86	**9.34E-03**	9.91E-01	-0.06 ± 0.08	-0.71	2.48E-01	7.52E-01
	Passive & Vibrations	**All**	18	**-0.30 ± 0.13**	-2.34	**1.58E-02**	9.84E-01	-0.11 ± 0.11	-0.96	1.76E-01	8.24E-01
	Active & Vibrations	**All**	15	**-0.17 ± 0.11**	-1.49	**7.94E-02**	9.21E-01	0.07 ± 0.07	1.06	8.47E-01	1.53E-01
	Active & Passive	**All**	13	**-0.43 ± 0.08**	-5.11	**1.29E-04**	1.00E+00	**-0.17 ± 0.06**	-2.98	**5.76E-03**	9.94E-01

Supplementary Table 6. Rostrocaudal spatial analysis for participant level results.

Task			Spinal levels (fMRI) / Roots stimulated (Intraoperative)						
Analysis	Condition (s)	Muscle	L1	L2	L3	L4	L5	S1	S2
**Participant -Specific** Second Level	Active	Gas	6.10%	7.92%	7.66%	12.98%	16.54%	28.38%	20.42%
		TA	6.80%	0.00%	17.11%	14.45%	43.16%	0.00%	18.48%
		Qd	25.80%	14.67%	0.00%	22.03%	37.51%	0.00%	0.00%
	Passive	Gas	20.77%	29.42%	23.80%	0.00%	0.00%	0.00%	26.02%
		TA	0.00%	5.70%	0.00%	12.78%	16.42%	15.61%	49.48%
		Qd	17.56%	34.35%	17.21%	0.00%	0.00%	0.00%	30.88%
		Il	7.84%	52.46%	13.99%	0.00%	25.71%	0.00%	0.00%
	Vibrations	Gas	25.95%	16.79%	11.25%	12.56%	0.00%	0.00%	33.46%
		TA	13.09%	9.96%	14.10%	21.90%	12.24%	12.77%	15.95%
		Qd	15.79%	13.16%	7.10%	26.93%	16.76%	0.00%	20.27%
		Il	12.78%	5.36%	17.30%	17.48%	9.30%	15.81%	21.97%
		BF	31.66%	21.25%	23.87%	23.22%	0.00%	0.00%	0.00%
		GMax	29.47%	31.49%	0.00%	39.04%	0.00%	0.00%	0.00%
**Participant -Specific** Third Level	Active & Passive & Vibrations	Gas	23.13%	32.20%	44.66%	0.00%	0.00%	0.00%	0.00%
	Active & Passive & Vibrations	TA	11.76%	0.00%	22.03%	35.38%	30.84%	0.00%	0.00%
	Active & Passive & Vibrations	Qd	18.36%	23.53%	0.00%	58.11%	0.00%	0.00%	0.00%
	Passive & Vibrations	Il	11.16%	43.43%	0.00%	18.84%	26.57%	0.00%	0.00%
Literature	Intraoperative stimulations	Gas	0.00%	0.40%	2.10%	11.10%	24.30%	29.30%	32.70%
		TA	0.00%	1.50%	6.60%	25.70%	34.10%	22.60%	9.50%
		Qd	4.90%	21.40%	33.30%	29.60%	9.80%	0.80%	0.20%
		Il	32.60%	44.20%	19.70%	2.60%	0.40%	0.40%	0.00%
		BF	0.00%	0.00%	6.70%	23.30%	40.00%	30.00%	0.00%
		GMax	4.30%	0.00%	0.50%	5.00%	24.10%	28.10%	38.10%

Supplementary Table 7. Personalized projectomes descriptions

Muscle			QUAD	IL	GAS	TA	BF	GMax	Total
Number of times identified			8	10	10	9	8	7	52
Ventral			2	7	5	6	3	4	27
Dorsal			6	3	5	3	5	3	25
Left			0	2	2	0	3	2	9
Right			8	8	8	9	5	5	43
L1			0	2	0	0	0	0	2
L2			3	5	0	0	0	0	8
L3			3	3	0	1	3	0	10
L4			1	0	1	3	1	3	9
L5			1	0	1	2	2	2	8
S1			0	0	2	1	2	0	5
S2			0	0	6	2	0	2	10
Total Corrected			3	4	3	2	0	0	12
Total Uncorrected			5	6	7	7	8	7	40
Participant-Specific Third Level	Active & Passive & Vibrations	Total	2	0	2	4	0	0	8
		Corrected	2	0	0	2	0	0	4
		Uncorrected	0	0	2	2	0	0	4
	Active & Vibrations	Total	0	0	3	2	0	0	5
		Corrected	0	0	1	0	0	0	1
		Uncorrected	0	0	2	2	0	0	4
	Passive & Vibrations	Total	2	6	0	1	0	0	9
		Corrected	1	3	0	0	0	0	4
		Uncorrected	1	3	0	1	0	0	5
	Active & Passive	Total	1	0	4	2	0	0	7
		Corrected	0	0	2	0	0	0	2
		Uncorrected	1	0	2	2	0	0	5
	Total 3rd Level	Total	5	6	9	9	0	0	29
		Corrected	3	3	3	2	0	0	11
		Uncorrected	2	3	6	7	0	0	18
Participant-Specific Second Level	Active	Total	0	0	0	0	0	0	0
		Corrected	0	0	0	0	0	0	0
		Uncorrected	0	0	0	0	0	0	0
	Passive	Total	0	2	0	0	0	0	2
		Corrected	0	1	0	0	0	0	1
		Uncorrected	0	1	0	0	0	0	1
	Vibrations	Total	3	2	1	0	8	7	21
		Corrected	0	0	0	0	0	0	0
		Uncorrected	3	2	1	0	8	7	21
	Total 2nd Level	Total	3	4	1	0	8	7	23
		Corrected	0	1	0	0	0	0	1
		Uncorrected	3	3	1	0	8	7	22

Supplementary Table 8. Participant description

	Count	Age (mean) [year]	Age (se) [year]	Height (mean) [cm]	Height (se) [cm]	Weight (mean) [kg]	Weight(se) [kg]
F	5	26.0	0.89	166.6	3.12	60.6	2.66
M	7	26.9	0.77	178.7	2.88	67.9	1.64
Total	12	26.5	0.57	171.0	2.71	58.5	1.76
